# Phytochemical analysis in some Iranian *Scutellaria* spp., a chemotaxonomic approach

**DOI:** 10.1038/s41598-026-51091-z

**Published:** 2026-05-13

**Authors:** Faezeh Golizadeh, Fatemeh Nejadhabibvash, Ahad Hedayati, Mozhgan Larti

**Affiliations:** 1 Department of Biology, Faculty of Science, Urmia University, Urmia, Iran; 2 Secondary Metabolite Production in Biological System Research Department, Iranian Academic Center for Education, Culture and Research (ACECR), West Azerbaijan Branch, Urmia, Iran; 3 West Azerbaijan Agricultural and Natural Resources Research and Education Centre, Urmia, Iran

**Keywords:** Chemical diversity, Essential oil, Fatty acid, Iran, Microbiology, Plant sciences

## Abstract

Species of the genus *Scutellaria* (Lamiaceae) are widely distributed and have long been used in traditional medicine for the treatment of gastrointestinal, cardiovascular, neurological, and respiratory disorders. However, comparative chemotaxonomic assessments of Iranian *Scutellaria* taxa remain limited. In this study, we investigated the phytochemical diversity and chemotaxonomic relationships among six Iranian *Scutellaria* species (including *Scutellaria pinnatifida* A. Hamilt., *Sc. araxensis* Grossh.*, Sc. bornmulleri* Hausskn. ex Bornm., *Sc. tomentosa* Bertol.*, Sc. theobromina* Rech F*.,* and *Sc. sosnowskyi* Grossh.) collected from natural habitats in Northwestern Iran, using integrated analyses of essential oils, phenolic compounds and fatty acid profiles. Substantial interspecific variation was detected across all metabolite classes, demonstrating pronounced biochemical heterogeneity within the genus. Essential oil analysis showed that α-pinene was most abundant in *Sc. araxensis* (21.64%) and *Sc. tomentosa* (14.43%), whereas trans-caryophyllene predominated in *Sc. bornmuelleri* (25.68%) and *Sc. theobromina* (25.07%). Germacrene D was the principal constituent of *Sc. pinnatifida* (40.16%), while *Sc. tomentosa* exhibited the highest caryophyllene oxide content (19.33%). High performance liquid chromatography (HPLC) profiling further revealed marked differences in phenolic composition, with gallic acid ranging from 11.59 to 31.54 mg/100 g DW, caffeic acid from 2.32 to 18.93 mg/100 g DW, chlorogenic acid from 1.10 to 23.96 mg/100 g DW, and rutin from 10.13 to 22.23 mg/100 g DW. Fatty acid composition also showed clear species-specific patterns: palmitic acid was highest in *Sc. bornmuelleri* (19.62%) and *Sc. araxensis* (19.40%), whereas oleic acid reached maximum levels in *Sc. sosnowskyi* (48.29%) and *Sc. tomentosa* (47.31%). Linoleic acid was most abundant in *Sc. tomentosa* (34.36%) and *Sc. sosnowskyi* (32.48%). Hierarchical clustering analysis (HCA) separated the taxa into three distinct groups, while principal component analysis (PCA) confirmed clear biochemical differentiation, with the first two components explaining most of the total variance. The strong concordance between both multivariate approaches supports the robustness of the inferred relationships. Overall, these findings demonstrate that integrated metabolite profiling is a powerful chemotaxonomic tool for species discrimination, biodiversity evaluation, and identification of promising *Scutellaria* germplasm for medicinal and industrial exploitation.

## Introduction

The Lamiaceae family contains 360 species of perennial flowering plants from the genus *Scutellaria* L., which is endemic to Asian countries, North America and Europe^1–3^. In Iran, 27 species have been identified, of which 12 are endemic^4^. *Scutellaria* species have long been utilized in traditional medicine and have recently attracted considerable attention due to their pharmacological potential. Clinical studies have explored their use as therapeutic or adjunct agents in the management of breast and prostate cancers, demonstrating low toxicity across various dosage forms. Traditionally, these species have been used for the treatment of various diseases, including diarrhea, hypertension, insomnia, respiratory infections, and neurological disorders such as anxiety and hysteria^2,5,6^. Phytochemical investigations have revealed that essential oils of *Scutellaria* species are rich in bioactive compounds, including germacrene D, β-caryophyllene, linalool, β-farnesene, and eugenol^7–12,5,13,6,14,15^. The composition of these essential oils has proven to be a valuable tool in resolving taxonomic relationships within the Lamiaceae family^16,17^. Notably, trans-caryophyllene and its derivatives, which are commonly detected in this genus, exhibit strong binding affinity to cannabinoid receptor type 2, implicating them in the modulation of physiological processes such as inflammation, pain, mood, arthrosclerosis and etc.^18^. In addition, caryophyllene oxide, an oxygenated sesquiterpenoid, has been widely recognized for its protective roles in pharmaceutical and nutraceutical applications, owing to its diverse biological activities, including anti-inflammatory, antifungal, analgesic, and anticholinesterase effects^19,20^. Flavonoids represent another major class of secondary metabolites in *Scutellaria*, contributing significantly to their antioxidant, anticancer, antimicrobial, and immunomodulatory properties^21–24^. Other phytochemical constituents, such as phytosterols, polysaccharides, and alkaloids, have also been reported^25^. Previous studies have documented the occurrence of flavonoids and phenolic acids in several *Scutellaria* taxa: *Sc. baicalensis*^26–29^, *Sc. albida*^30^, *Sc. pinnatifida* subsp *alpina*^31^, *Sc. incana*^32^, *Sc. radix*^33^ and *Sc. altissima* L. *Sc. farsistanica*, *Sc. tomentosa*, *Sc. pinnatifida* subsp. *pichleri*, *Sc. patonii*, *Sc. multicaulis* var. *multicaulis*, *Sc. nepetifolia* & *Sc. condensata* subsp. *condensata*^34^. In addition, flavonoid compounds have been proven to be of chemotaxonomic importance^35–44^. Compounds such as gallic acid, caffeic acid and rutin are of particular interest due to their well-documented pharmacological activities, including antioxidant, anti-inflammatory, and anticancer effects. These metabolites have been shown to inhibit cancer cell proliferation and exhibit therapeutic potential in a variety of chronic diseases^45–48^. Furthermore, fatty acid composition has been recognized as a reliable chemotaxonomic marker in higher plants, providing additional insight into species differentiation^49–59^. To the best of our knowledge, previous studies on these taxa have been limited in scope, fragmented, or primarily focused on isolated phytochemical, pharmacological, or floristic aspects. No comprehensive study has comparatively assessed these endemic species within an integrated chemotaxonomic framework using multivariate approaches. Given the ecological and medicinal importance of *Scutellaria* species in Iran, as well as their potential pharmaceutical and nutraceutical applications, the present study aimed to investigate the chemical diversity and chemotaxonomic relationships of selected Iranian *Scutellaria* species, with particular emphasis on phenolic compounds, fatty acids, and essential oil composition.

## Material and methods

### Plant material

**Fig. 1:**
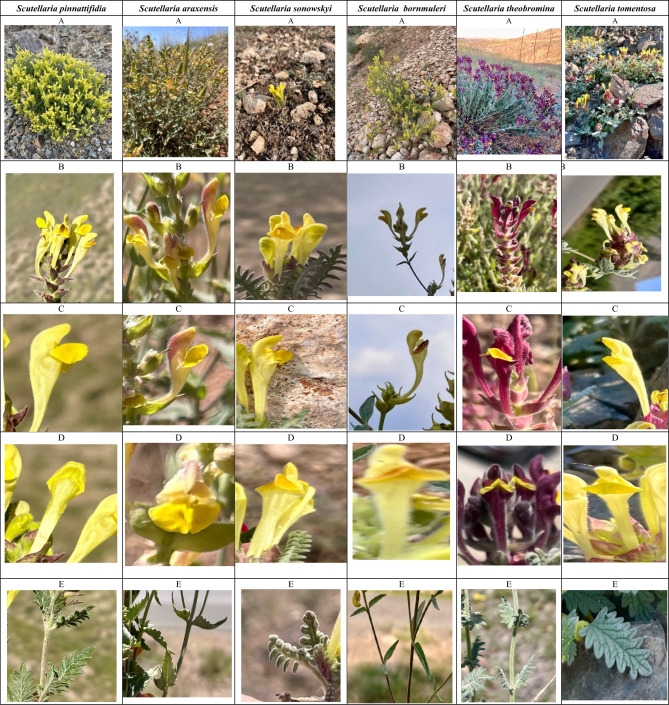
(**A**). Habitus and habitat, (**B**). inflorescence, (**C**). lateral view of flower (**D**). frontal view of flower, (**E**). leaves.

The Aerial parts of investigated *Scutellaria* L. species including *Scutellaria pinnatifida* A. Hamilt., *Sc. araxensis* Grossh.*, Sc. bornmulleri* Hausskn. ex Bornm., *Sc. tomentosa* Bertol.*, Sc. theobromina* Rech F*.,* and *Sc. sosnowskyi* Grossh., were collected at the complete flowering stage during May to July 2025 from natural habitats in the Northwest of Iran (Table 1). Some of plants harvested for seed oil extraction and fatty acid analysis at fruiting stage during July to 2025. The collection was conducted in line with relevant institutional, national and international guidelines, including the IUCN Policy Statement on Research Involving Species at Risk of Extinction and the Convention on International Trade in Endangered Species of Wild Fauna and Flora. The taxonomic identification is authenticated by Dr. Larti, Member of Scientific Board of Agriculture and Natural Resources Research and Education Center of West Azerbaijan Province, Urmia, Iran, and plant voucher specimens were placed at the Herbarium of the West-Azerbaijan Agricultural and Natural Resources Research and Education Center, Urmia, Iran. The herbarium codes and their collection sites are illustrated in Table 1. Pictures of the 6 *Scutellaria *species investigated in this study, were presented in Fig. 1.

**Table 1:** Coordination and location of *Scutellaria* L. species collected.

Scientific names	Locality	Geographical location	Voucher number
*Scutellaria pinnatifida* A. Hamilt.	Ardebil Province: Arasbaran protected area	38°54′17.39"N46°51′32.39"E	11,079
*Scutellaria araxensis* Grossh.	West Azerbaijan Province: Urmia: Qushchi	38.03°58′ 21″N45.12°05′09″E	11,078
*Scutellaria bornmuelleri* Hausskn. ex Bornm.	West Azerbaijan Province: Chaldoran	39.14°49′36″N44.69°44′57″E	11,080
*Scutellaria tomentosa* Bertol.	East Azerbaijan Province: Khomarlu road: 70 km to Kaleibar	38.89°42′41″N46.40°53′8″E	11,077
*Scutellaria sonowskyi* Grossh.	West Azerbaijan Province: Chaldoran: Qareh Kelisa Road	39.06°53′2″N44.57°32′44″E	11,082
*Scutellaria theobromina* Rech F.	West Azerbaijan Province: Urmia: Nushin Shahr	37.94°41′1″N44.95°5′42″E	11,081

### The essential oil extraction

The aerial parts of the plants were dried at room temperature and hydrodistilled for 3 h using a Clevenger-type apparatus. The volatile oil was dried by (Na_2_SO_4_) and held in the closed dark vial at 4 °C before the analysis^10^. The EO was extracted at Secondary Metabolite Production in Biological System Research Department, Iranian Academic Center for Education, Culture and Research (ACECR), West Azerbaijan Branch, Urmia, Iran.

### Essential oil analysis

Essential oils were evaluated using GC–MS. For GC–MS analysis an Agilent 7890 A gas chromatograph coupled to a 5975C mass spectrometer using a HP-5 MS capillary column (5% Phenyl Methylpolysiloxane, 30 m length, 0.25 mm i.d., 0.25 μm film thickness) was used. The oven temperature was programmed as follows: 3 min at 80 °C, thereafter, 8 °C min^−1^ to 180 °C, held for 10 min at 180 °C. Helium was used as carrier gas at a flow rate of 1 mL min^-1^ and Electron impact (EI) was 70 eV. The injector was set in a split mode (split ratio of 1:500) and mass range acquisition was from 40 to 500 m/z. The essential oil constituents were identified by comparison their retention indices mass spectra fragmentation with those in a stored Wiley 7n.1 mass computer library and those of the National Institute of Standards and Technology (NIST)^60^. Relative percentage amounts were calculated from peak area using a Shimadzu C R4A chromatopac.

### Analysis of secondary metabolites (HPLC)

#### Preparation of the methanolic extract

The leaves of *Scutellaria* species growing naturally in Northwest of Iran were shade dried at 25 °C, and the Plant materials were ground and prepared for extraction. To extract polyphenolic compounds, 0.8 g of each plant species was crushed with liquid nitrogen and 5 ml of 80% methanol was immediately added to it. Then, the resulting mixture was placed in an ultrasonic device for 20 min at a temperature of 25 °C^61^. The resulting methanol extract was centrifuged for 10 min at 2000 rpm and, after passing through a 0.22 µm, was injected into an HPLC device for analysis of the compounds.

#### Identification of metabolites

The separation, identification, and quantitative determination of the polyphenolic compounds studied in this study were performed using an HPLC model 1100 series manufactured by Agilent, USA, equipped with a 20 µl injection loop, a four-solvent gradient pump, and a diode array detector. Separation was performed on an octadecylsilane column (25 cm long, 4.6 mm internal diameter, and 5 μm particle size (Shimadzu Kyoto, Japan). The standard curve was also drawn based on the area under the standard curve of all compounds (Sigma products) at concentrations of 5, 10, 25, 50, 100, 250, and 500 mg/ml. The solvent flow rate was 1 mL/min and the mobile phase consisted of acetonitrile and acetic acid buffer in ratios of 10:80. Choosing the appropriate wavelength is one of the important points in the analysis of compounds, and considering that the compounds analyzed were different from the wavelengths of 250 nm (rosmarinic acid), 272 nm (gallic acid), 310 nm (caffeic acid), 320 nm (chlorogenic acid), 310 nm (coumaric acid), 272 nm (cinnamic acid), 360 nm (quercetin) and 310 nm (rutin) were used. The compounds were identified qualitatively by comparing the retention times obtained from the chromatographic settings of the tested samples with those of known standards. The determined amounts of analytes were presented as “content “(expressed in mg/g 100 g DW)^62^.

### Fatty acids analysis

Total lipids extracted from seeds of *Scutellaria* species^63^. The formation of FAME (Fatty Acid Methyl Esters) was carried out according to the procedure described by Desvilettes et al. (1994). The sample was saponified with methanolic sodium hydroxide and the fatty acids were esterified with methanolic sulfuric acid. FAME were analyzed with a 6890 N GC–FID (Agilent Technologies, Wilmington, DE, USA) fitted with a J&W DB-Wax capillary column (30m, 0.25 mm id., 0.25 mm film thickness), a split– splitless injector with Agilent tapered liner (4mm id) and flame ionization detector. The initial column temperature was maintained at 100°C for 1 min and then raised at 25°C/min to 190°C and held for 10 min and then raised to 220°C and held for 15 min. Nitrogen was used as carrier and makeup gas, at flow rates of 1.0 and 45 mL/min, respectively. The injector and detector temperature were held at 250 and 260°C, respectively. Chem. Station software was used for online data collection and processing^64^. Individual FAME was identified by comparison to known standards (Sigma, Chemical Co. St. Louis). Fatty acids were recognized by comparison with synthetic standards.

### Statistical analysis

The active constituents in the plant species were used as markers for chemotaxonomy. Data were analyzed by one-way ANOVA using SPSS software, Version 23. Results were tested for significance by using Duncan’s range test. This test was to evaluate the differences among species. Each experiment consisted of three replicate samples of each of 6 *Scutellaria* species injected into the analytical instrument. The degree of association between the two variables was determined by calculating Pearson’s correlation coefficient. Hierarchical clustering analysis (HCA) of main components between species was performed with average linkage method and the resulting dendrogram was illustrated. Principal component analysis (PCA) was performed to reduce data dimensionality and identify major patterns of variation among species. All values where p ≤ 0.05 were considered statistically significant.

## Results and discussion

### Estimation of active constituents

The chemical constituent pattern of six species of *Scutellaria*; *Sc. pinnatifida Sc. araxensis, Sc. bornmulleri, Sc. tomentosa, Sc. theobromina* and *Sc. sonowskyi* were surveyed and compared.

**Fig. 2:**
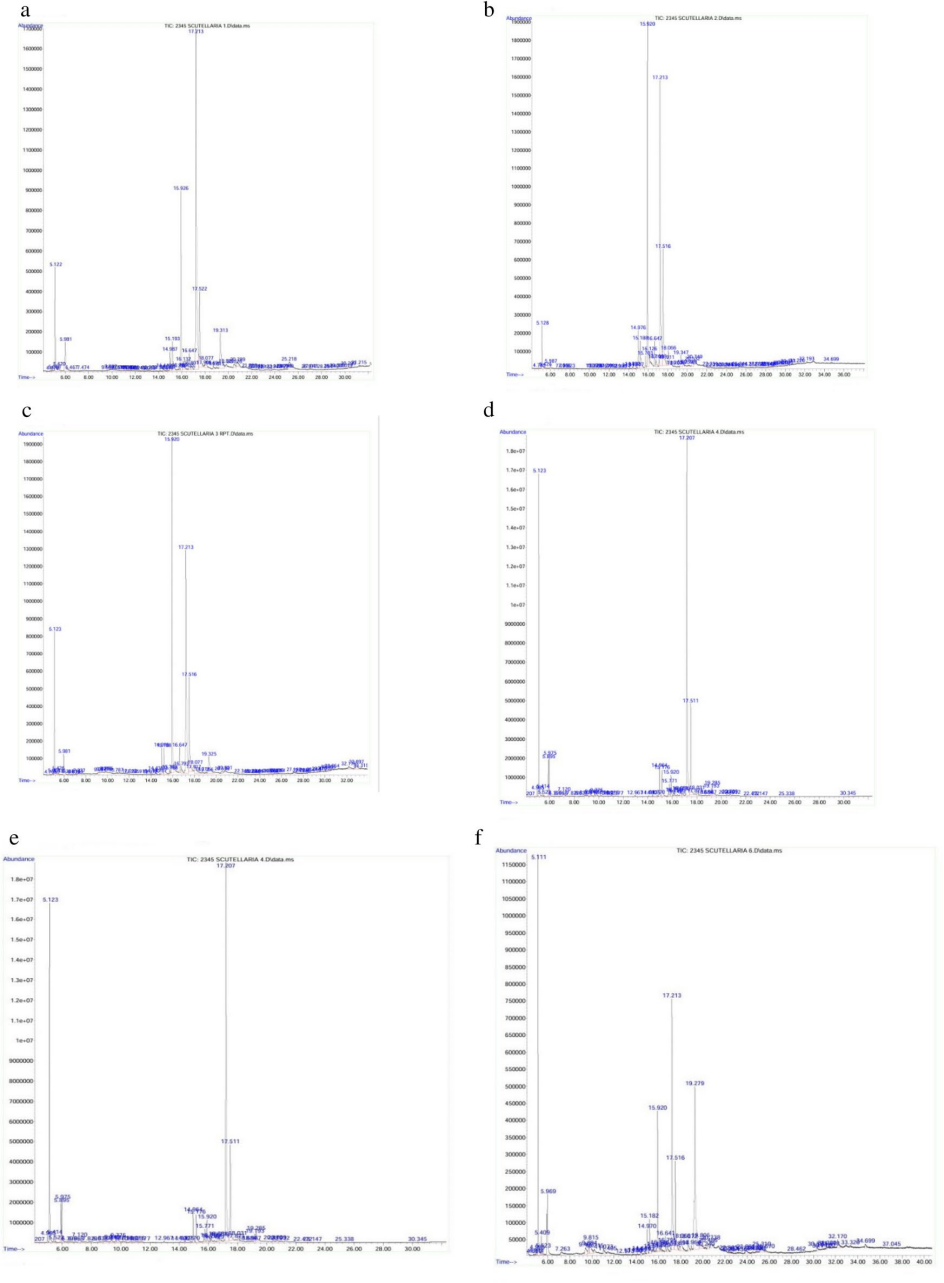
(**a**): GC–MS Chromatogram of *Sc. sonowskyi* EO. (**b**): GC–MS Chromatogram of *Sc. bornmulleri* EO. (**c**): GC–MS Chromatogram of *Sc. theobromina* EO. (**d**): GC–MS Chromatogram of *Sc. araxensis* EO. (**e**): GC–MS Chromatogram of *Sc. pinnatifida* EO. (**f**): GC–MS Chromatogram of *tomentosa* EO.

The essential oil compositions of the *Scutellaria* species are listed in Table 2, in which the percentage and retention indices of the total components are given. GC–MS chromatograms of all six species are presented in Fig. 2a-2f. Eight compounds constituting about 74.30% of the essential oil of *Sc. tomentosa*, 8 compounds constituting 86.13% of the oil of *Sc. bornmulleri*, 8 compounds constituting 84.97% of the oil of *Sc. pinnatifida*, 8 compounds constituting 81.58% of the oil of *Sc. sonowskyi*, 8 compounds constituting 83.06% of the oil of *Sc. araxensis* and 8 compounds constituting 86.13% of the oil of *Sc. theobromina* were characterized (Table 2).

**Table 2:** Constituents identified in the essential oil of the aerial parts of *Scutellaria* species.

Species	Constituents	Content (%)^b^	RI^a^	Species	Constituents	Content (%)^b^	RI
*Sc. tomentosa*	Sabinene	5.18a	976	*Sc. sonowskyi*	Sabinene	3.51b	976
	α- Pinene	14.43b	939		α- pinene	6.23c	939
	β-Bourbonene	2.47e	1384		β-Bourbonene	3.41b	1384
	Trans- Caryophyllene	7.24e	1416		Trans- Caryophyllene	12.33d	1416
	α-Copaene	1.38f	1376		α-Copaene	1.63e	1376
	Germacrene D	18.15f	1480		Germacrene D	36.44c	1480
	Caryophyllene oxide	19.33a	1583		Caryophyllene oxide	8.33b	1583
	α-Cubenene	6.12f	1391		α-Cubenene	9.70c	1391
	Phytochemical group				Phytochemical group		
	Monoterpene hydrocarbon	19.61			Monoterpene hydrocarbon	9.74	
	Sesquiterpene hydrocarbons	35.36			Sesquiterpene hydrocarbons	63.51	
	Oxygenated sesquiterpenes	19.33			Oxygenated sesquiterpenes	8.33	
	Total	74.3			Total	81.58	
*Sc. bornmulleri*	Sabinene	2.20d	976	*Sc. araxensis*	Sabinene	3.14c	976
	α- pinene	3.24f	939		α- pinene	21.64a	939
	β-Bourbonene	3.51a	1384		β-Bourbonene	3.17d	1384
	Trans- Caryophyllene	25.68a	1416		Trans- Caryophyllene	2.03f	1416
	α-Copaene	3.53a	1376		α-Copaene	2.62b	1376
	Germacrene D	32.29d	1480		Germacrene D	38.55b	1480
	Caryophyllene oxide	2.44e	1583		Caryophyllene oxide	2.01f	1583
	α-Cubenene	13.24b	1391		α-Cubenene	9.91d	1391
	Phytochemical group				Phytochemical group		
	Monoterpene hydrocarbon	5.44			Monoterpene hydrocarbon	24.78	
	Sesquiterpene hydrocarbons	78.25			Sesquiterpene hydrocarbons	56.27	
	Oxygenated sesquiterpenes	2.44			Oxygenated sesquiterpenes	2.01	
	Total	86.13			Total	83.06	
*Sc. pinnatifida*	Sabinene	0.87e	976	*Sc. theobromina*	Sabinene	0.54f	976
	α- pinene	5.23e	939		α- pinene	8.7c	939
	β-Bourbonene	2.13f	1384		β-Bourbonene	3.23c	1384
	Trans- Caryophyllene	16.15c	1416		Trans- Caryophyllene	25.07b	1416
	α-Copaene	2.42c	1376		α-Copaene	2.41d	1376
	Germacrene D	40.16a	1480		Germacrene D	26.79e	1480
	Caryophyllene oxide	4.35c	1583		Caryophyllene oxide	4.24d	1583
	α-Cubenene	13.66a	1391		α-Cubenene	10.53c	1391
	Phytochemical group				Phytochemical group		
	Monoterpene hydrocarbon	6.21			Monoterpene hydrocarbon	9.24	
	Sesquiterpene hydrocarbons	74.41			Sesquiterpene hydrocarbons	68.03	
	Oxygenated sesquiterpenes	4.35			Oxygenated sesquiterpenes	4.24	
	Total	84.97			Total	81.51

The present study revealed that germacrene D, trans-caryophyllene, α-pinene and caryophyllene oxide represent the principal constituents of the essential oils obtained from the investigated *Scutellaria* species, while the overall predominance of sesquiterpene hydrocarbons across taxa suggests a broadly conserved chemotaxonomic signature within the genus (Table 2). Despite this shared pattern, substantial interspecific variation was evident in the relative abundance of individual metabolites, indicating marked divergence in volatile secondary metabolism among species. Among the major monoterpenes, α-pinene was particularly enriched in *Sc. araxensis* (21.64%) and *Sc. tomentosa* (14.43%), whereas markedly lower proportions were detected in *Sc. pinnatifida* (5.23%) and *Sc. sonowskyi* (6.23%) (Table 2). Similarly, trans-caryophyllene showed pronounced variability, ranging from 2.03% to 25.68%, with the highest levels recorded in *Sc. bornmuelleri* (25.68%) and *Sc. theobromina* (25.07%), and the lowest in *Sc. araxensis* (2.03%). Germacrene D also varied considerably among species, from 18.15% to 40.16%, reaching its maximum in *Sc. pinnatifida* and minimum in *Sc. tomentosa*. In contrast, caryophyllene oxide accumulated most strongly in *Sc. tomentosa* (19.33%), whereas notably lower levels were observed in *Sc. bornmuelleri* (2.44%) and *Sc. araxensis* (2.01%) (Table 2). Collectively, these species-specific compositional shifts suggest differential regulation of terpene biosynthetic pathways and may reflect both genetic divergence and ecological adaptation.

Correlation analysis further highlighted coordinated and antagonistic relationships among volatile metabolites (Table 3). Strong positive associations were observed between sabinene and caryophyllene oxide (r = 0.79), α-cubenene and α-copaene (r = 0.76), germacrene D and α-cubenene (r = 0.69), and sabinene with α-pinene (r = 0.61). These positive relationships may indicate shared biosynthetic precursors, co-regulated enzymatic steps, or parallel ecological functions. Conversely, strong negative correlations were detected between sabinene and α-cubenene (r =  − 0.98), sabinene and α-copaene (r =  − 0.83), caryophyllene oxide and α-pinene (r =  − 0.80), α-cubenene and caryophyllene oxide (r =  − 0.81), α-copaene and caryophyllene oxide (r =  − 0.79), and germacrene D with caryophyllene oxide (r =  − 0.75) as well as caryophyllene oxide and sabinene (-0.69), followed by α-pinene and α-cubenene (-0.60) among others (Table 3). Such antagonistic patterns may reflect competition for common terpene precursors or shifts in pathway flux toward alternative end products.

**Table 3:** Correlation coefficients among essential oil compounds using GC–MS on studied *Scutellaria* species.

Standards	α-Pinene	Sabinene	α-Copaene	Bourbonene	Trans-caryophyllene	Germacrene D	α-Cubenene	Caryophyllene oxide
α-Pinene	1	0.61**	– 0.25^ ns^	– 0.08^ ns^	– 0.80**	– 0.12^ ns^	– 0.60**	0.17^ ns^
Sabinene	0.61**	1	– 0.83**	– 0.11^ ns^	-0.69**	– 0.56*	– 0.98**	0.79**
α-Copaene	– 0.25^ ns^	-0.83**	1	0.38^ ns^	0.52*	0.39^ ns^	0.76**	– 0.79**
Bourbonene	– 0.08^ ns^	– 0.11^ ns^	0.38^ ns^	1	0.28^ ns^	0.09^ ns^	0.07^ ns^	– 0.41^ ns^
Trans-Caryophyllene	– 0.80**	– 0.69**	0.52*	0.28^ ns^	1	– 0.07^ ns^	0.59**	– 0.36^ ns^
Germacrene	– 0.12^ ns^	– 0.56*	0.39^ ns^	0.09^ ns^	– 0.07^ ns^	1	0.69**	– 0.75**
α-Cubenene	– 0.60**	– 0.98**	0.76**	0.07^ ns^	0.59**	0.69**	1	– 0.81**
Caryophyllene Oxide	0.17^ ns^	0.79**	-0.79**	– 0.41^ ns^	– 0.36^ ns^	– 0.75**	– 0.81**	1

^a^Retention Index, ^b^ percentage concentration, Compounds in bold represent the major constituents. Means within each column followed by the same letter are not different according to the Duncan test.

*Correlation is significant at the 0.05 level. **Correlation is significant at the 0.01 level. ns. Not significant.

Previous studies have documented substantial chemical diversity in *Scutellaria* essential oils, with reported major constituents including hexadecanoic acid, germacrene D, β-caryophyllene, linalool, β-farnesene, eugenol, anethole, carvacrol, cadinene, and scutellarine^65,13,6,15,67,68^. Consistent with these earlier reports^69,70,5^, the present data confirm that sesquiterpenes and monoterpenes remain dominant chemical classes in the genus. However, the relative enrichment of germacrene D, caryophyllene oxide, trans-caryophyllene, and α-pinene observed here underscores considerable quantitative heterogeneity among species. This variability is likely driven by multiple interacting factors, including genotype, geographical origin, altitude, edaphic conditions, water availability, developmental stage, and phenological timing of harvest, all of which are known to influence essential oil biosynthesis. From an applied perspective, these findings provide a basis for selecting *Scutellaria* taxa with desirable volatile profiles for pharmaceutical, nutraceutical, cosmetic or agro-industrial applications. For example, species enriched in α-pinene or caryophyllene derivatives may be valuable sources of bioactive terpenes with reported antimicrobial, anti-inflammatory or antioxidant properties. In addition, the compositional differentiation identified among taxa supports the utility of volatile markers in future chemotaxonomic studies of the genus.

**Fig. 3:**
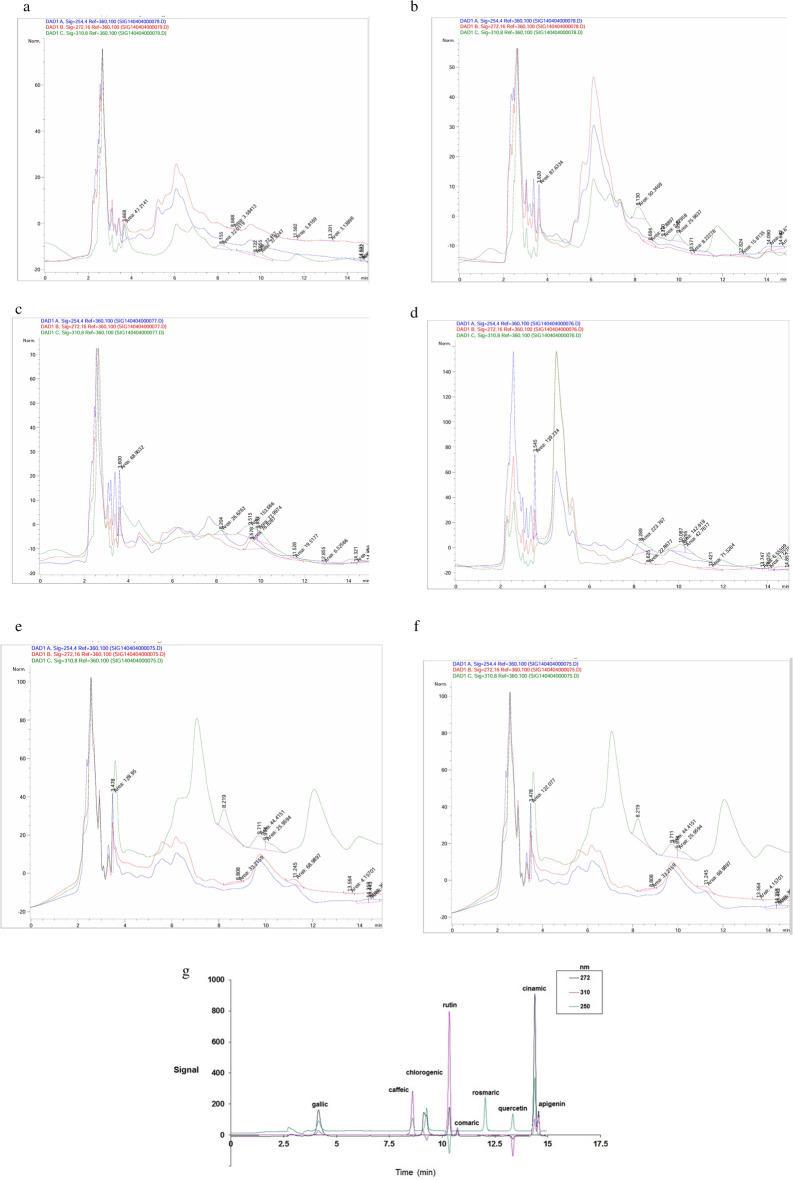
(**a**): HPLC Chromatogram of *Sc. bornmulleri* phenolic compounds. (**b**): HPLC Chromatogram of *Sc. theobromina* phenolic compounds. (**c**): HPLC Chromatogram of *Sc. araxensis* phenolic compounds. (**d**): HPLC Chromatogram of *Sc. pinnatifida* phenolic compounds. (**e**): HPLC Chromatogram of *Sc. tomentosa* phenolic compounds. (**f**): HPLC Chromatogram of *Sc. sonowskyi* phenolic compounds. (**g**): Chromatogram of HPLC standards.

Leaf extracts of the investigated species exhibited substantial variation in both the composition and concentration of phenolic compounds, as determined by HPLC analysis (Tables 4 and 5). Representative chromatograms for each of the six studied species are included in Figs. 3a-3f. Based on comparisons with available reference standards, nine phenolic compounds were identified, and their corresponding calibration curves are shown in Fig. 3 g.

**Table 4:** Major phenolic and flavonoid compounds of the studied *Scutellaria* species based on HPLC analysis.

Species	Name of compounds	Content ^a^(mg/100 g DW)	Species	Name of compounds	Content (mg/100 g DW)
*Sc. tomentosa*	Gallic acid	**31.54**b	*Sc. sonowskyi*	Gallic acid	**29.88**c
	Caffeic acid	4.81b		Caffeic acid	4.81b
	Chlorogenic acid	**10.40**b		Chlorogenic acid	**10.39**b
	*p*-Coumaric acid	1.41c		*p*-Coumaric acid	1.42c
	Rutin	**13.52**d		Rutin	**13.53**e
	Rosmarinic acid	3.80b		Rosmarinic acid	3.88b
	Quercetin	0.89d		Quercetin	0.90c
	Cinnamic acid	0.92b		Cinnamic acid	0.90b
	Apigenin	5.69a		Apigenin	5.72a
*Sc. bornmulleri*	Gallic acid	**11.59**f	*Sc. araxensis*	Gallic acid	**16.80**e
	Caffeic acid	2.71d		Caffeic acid	2.32e
	Chlorogenic acid	1.10e		Chlorogenic acid	**23.96**a
	*p*-Coumaric acid	0.70d		*p*-Coumaric acid	4.66a
	Rutin	**19.59**b		Rutin	**10.13**f
	Rosmarinic acid	0.31e		Rosmarinic acid	1.10c
	Quercetin	0.70e		Quercetin	1.42b
	Cinnamic acid	0.103d		Cinnamic acid	0.00e
	Apigenin	0.90d		Apigenin	0.61e
*Sc. pinnatifida*	Gallic acid	**34.28**a	*Sc. theobromina*	Gallic acid	**21.51**d
	Caffeic acid	**18.93**a		Caffeic acid	4.32e
	Chlorogenic acid	**7.22**d		Chlorogenic acid	7.83c
	*p*-Coumaric acid	4.41b		*p*-Coumaric acid	0.106e
	Rutin	**22.23**a		Rutin	**13.55**c
	Rosmarinic acid	4.00a		Rosmarinic acid	0.52d
	Quercetin	1.41b		Quercetin	3.30a
	Cinnamic acid	0.21c		Cinnamic acid	1.11a
	Apigenin	1.62c		Apigenin	5.12b

a: The values are expressed in mg/ 100g of sample dry weight. The data were sorted based on the retention time (RT) of components. Compounds in bold represent the major constituents. Means within each column followed by the same letter are not different according to the Duncan test.

The major polyphenolic constituents detected in the methanolic extracts of *Scutellaria* species included gallic acid, caffeic acid, chlorogenic acid, *p*-coumaric acid, rutin, rosmarinic acid, quercetin, cinnamic acid and apigenin (Table 4).

Quantitative analysis revealed considerable interspecific variation. Gallic acid content ranged from 11.59 to 34.28 mg/100 g dry weight (DW), with the highest concentration observed in *Sc. pinnatifida* and the lowest in *Sc. bornmulleri* (Table 4). Similarly, caffeic acid content varied between 2.32 and 18.93 mg/100 g DW, with maximum and minimum levels recorded in *Sc. pinnatifida* and *Sc. araxensis*, respectively (Table 4). Chlorogenic acid content showed a range of 1.10 to 23.96 mg/100 g DW, reaching its highest level in *Sc. araxensis* and lowest in *Sc. bornmulleri*. Rutin content also varied significantly among species, with the highest (22.23 mg/100 g DW) detected in *Sc. pinnatifida* and the lowest (10.13 mg/100 g DW) in *Sc. araxensis* (Table 4).

Comparable levels of rutin have previously been reported in *Scutellaria baicalensis*^71^, supporting the recurrent occurrence of this flavonoid across the genus. To our knowledge, the present study provides the first report of gallic acid and chlorogenic acid in methanolic extracts of the investigated *Scutellaria* species. In addition, the detection of apigenin is consistent with recent findings in *Sc. baicalensis*, further emphasizing the importance of flavonoids as major bioactive constituents within this genus^72^. The phytochemical profile observed here, characterized by the predominance of gallic acid and rutin in most analyzed species, differs from several previous reports in which baicalin, wogonin, verbascoside, and related flavonoids were identified as principal metabolites^73–75^. Other studies have likewise described diverse classes of specialized metabolites in *Scutellaria*, including flavones, flavanones, flavonols, chalcones, isoflavones, C-glucosyl flavones, isovitexin, flavone glycosides and phenylethanoid glycosides such as martynoside and verbascoside^76,77,75^. Collectively, these findings indicate pronounced phytochemical heterogeneity within the genus. Such variation is likely shaped by multiple interacting factors, including species-specific genetic background, environmental and climatic conditions, geographical origin, plant organ sampled, developmental stage at harvest, and differences in extraction or analytical methodology. Therefore, while the present results are broadly consistent with earlier studies highlighting the phenolic richness of *Scutellaria*, they also reveal substantial qualitative and quantitative variation in individual metabolites among species. This chemical diversity may have important taxonomic and applied implications. Variation in phenolic and flavonoid composition has long been considered a useful criterion for plant classification and species discrimination, and similar metabolite-based differentiation has been reported in several other genera, including *Salvia* spp. (Lamiaceae)^61^, *Eugenia* spp. (Myrtaceae), *Cotylelobium* spp. (Dipterocarpaceae)^78^, *Stemonoporus* spp. (Dipterocarpaceae)^78^, *Matricaria* L. (Asteraceae)^42^, and *Phlomis* L. (Lamiaceae)^37^. Accordingly, the phytochemical markers identified in this study may contribute to future chemotaxonomic frameworks for *Scutellaria* and may also support the selection of species with desirable metabolite profiles for pharmacological or nutraceutical applications. Nevertheless, the present study was limited to methanolic extracts and targeted compound analysis. Broader metabolomic approaches combined with multi-environment sampling would provide a more comprehensive understanding of chemical diversity and its ecological or genetic determinants within the genus. 

**Table 5:** Correlation coefficients among phenolic acid compounds using HPLC on studied *Scutellaria* species.

Standards	Gallic acid	Caffeic acid	Chlorogenic acid	*p*-Coumaric acid	Rutin	Rosmarinic acid	Quercetin	Cinnamic acid	Apigenin
Gallic Acid	1	0.66**	– 0.02^ ns^	0.21^ ns^	0.17^ ns^	0.93**	– 0.06^ ns^	0.42^ ns^	0.53*
Caffeic Acid	0.66**	1	– 0.23^ ns^	0.49*	0.71**	00.56*	0.01^ ns^	– 0.17^ ns^	– 0.16^ ns^
Chlorogenic Acid	– 0.02^ ns^	– 0.23^ ns^	1	0.62**	– 0.74**	0.02^ ns^	0.05^ ns^	– 0.20^ ns^	– 0.14^ ns^
*p*-Coumaric Acid	0.21^ ns^	0.49*	0.62**	1	0.06^ ns^	0.28^ ns^	– 0.22^ ns^	-0.67**	– 0.59**
Rutin	0.17^ ns^	0.71**	– 0.74**	0.06^ ns^	1	0.17^ ns^	– 0.20^ ns^	– 0.33^ ns^	– 0.35^ ns^
Rosmarinic Acid	0.93**	0.56*	0.02^ ns^	0.28^ ns^	0.17^ ns^	1	– 0.40^ ns^	0.25^ ns^	0.42^ ns^
Quercetin	– 0.06^n^	0.01^ ns^	0.05^ ns^	– 0.22^ ns^	-0.20^ ns^	– 0.40^ ns^	1	0.42^ ns^	0.22^ ns^
Cinnamic Acid	0.42^n^	– 0.17^ ns^	– 0.20^ ns^	– 0.67**	– 0.33^ ns^	0.25^ ns^	0.42^ ns^	1	0.97**
Apigenin	0.53*	– 0.16^ ns^	– 0.14^ ns^	– 0.59**	– 0.35^ ns^	0.42^ ns^	0.22^ ns^	0.97**	1

*Correlation is significant at the 0.05 level.

**Correlation is significant at the 0.01 level. ns. Not significant.

**Fig. 4:**
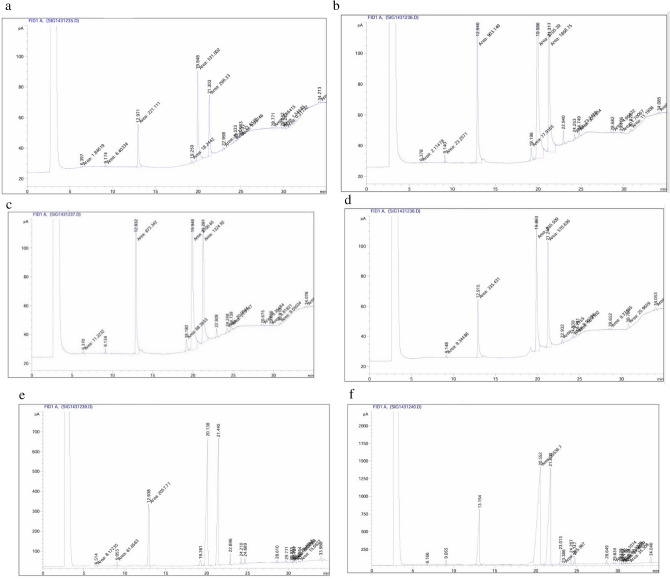
(**a**): GC Chromatogram of *Sc. bornmulleri* fatty acids. (**b**): GC Chromatogram of *Sc. theobromina* fatty acids. (**c**): GC Chromatogram of *Sc. araxensis* fatty acids. (**d**): GC Chromatogram of *Sc. pinnatifida* fatty acids. (**e**): GC Chromatogram of *Sc. tomentosa* fatty acids. (**f**): GC Chromatogram of *Sc. sonowskyi* fatty acids.

Gas chromatography (GC) analysis of crude oils obtained from the n-hexane fraction of the investigated *Scutellaria* species led to the identification of 11 bioactive constituents, including six saturated fatty acids (palmitic acid, stearic acid, myristic acid, behenic acid, lauric acid, and lignoceric acid) and five unsaturated fatty acids (oleic acid, linoleic acid, docosahexaenoic acid, cis-11-eicosaenoic acid, and nervonic acid) (Table 6; Fig. 4a–f.). Significant interspecific variation in fatty acid composition was observed among the studied *Scutellaria* species (p ≤ 0.05). Based on mean comparisons, the highest levels of palmitic acid were detected in *Sc. bornmulleri* (19.62%) and *Sc. araxensis* (19.40%). The highest oleic acid contents were found in *Sc. sonowskyi* (48.29%) and *Sc. tomentosa* (47.31%), while linoleic acid reached its maximum levels in *Sc. tomentosa* (34.36%) and *Sc. sonowskyi* (32.48%) (Table 6). Correlation analysis revealed strong positive relationships between oleic and linoleic acids (0.98), behenic acid and docosahexaenoic acid (0.86), oleic and palmitic acids (0.74), and palmitic and linoleic acids (0.66), followed by palmitic and lauric acids (0.62) (Table 7). The strongest negative correlation was observed between lauric acid and docosahexaenoic acid (− 0.63) (Table 7).

**Table 6:** Fatty acid composition of *Scutellaria* species.

Species	Name of compounds	Content (%)	Species	Name of Compounds	Content (%)
*Sc. tomentosa*	Lauric Acid	0.04c	*Sc. sonowskyi*	Lauric Acid	0.0093e
	Myristic Acid	0.33d		Myristic Acid	0.32e
	Palmitic Acid	**11.24b**		Palmitic Acid	**13.90b**
	Stearic Acid	1.71a		Stearic Acid	^n^0.00e
	Oleic Acid	**47.31a**		Oleic Acid	**48.29a**
	Linoleic Acid	**34.36a**		Linoleic Acid	**32.48a**
	Cis-11-Eicosenoic Acid	0.74b		Cis-11-Eicosenoic Acid	0.43d
	Behenic Acid	0.35c		Behenic Acid	0.66a
	Lignoceric Acid	0.47b		Lignoceric Acid	0.07d
	Nervonic Acid	^n^0.00c		Nervonic Acid	0.04b
	Docosahexaenoic Acid	0.67c		Docosahexaenoic Acid	0.82b
	ΣSFA	14.16		ΣSFA	14.98
	ΣUSFA	83.10		ΣUSFA	82.08
	Total	97.20		Total	97.06
*Sc. bornmulleri*	Lauric Acid	0.16b	*Sc. araxensis*	Lauric Acid	0.24a
	Myristic Acid	0.56a		Myristic Acid	0.32e
	Palmitic Acid	**19.62a**		Palmitic Acid	**19.40a**
	Stearic Acid	1.61b		Stearic Acid	1.51c
	Oleic Acid	**47.25a**		Oleic Acid	**46.71a**
	Linoleic Acid	**26.39a**		Linoleic Acid	**29.46b**
	Cis-11-Eicosenoic Acid	1.03a		Cis-11-Eicosenoic Acid	0.44d
	Behenic Acid	0.23a		Behenic Acid	0.20e
	Lignoceric Acid	^n^0.00e		Lignoceric Acid	0.20c
	Nervonic Acid	^n^0.00c		Nervonic Acid	^n^0.00c
	Docosahexaenoic Acid	0.55d		Docosahexaenoic Acid	0.22e
	ΣSFA	22.21		ΣSFA	21.91
	ΣUSFA	75.23		ΣUSFA	76.84
	Total	97.44		Total	98.75
*Sc. pinnatifida*	Lauric Acid	^n^0.00f	*Sc. theobromina*	Lauric Acid	0.03d
	Myristic Acid	0.50b		Myristic Acid	0.40c
	Palmitic Acid	6.05b		Palmitic Acid	**10.53b**
	Stearic Acid	^n^0.00e		Stearic Acid	1.36d
	Oleic Acid	**15.10b**		Oleic Acid	**15.80b**
	Linoleic Acid	**10.30b**		Linoleic Acid	**10.93b**
	Cis-11-Eicosenoic Acid	0.53c		Cis-11-Eicosenoic Acid	0.41d
	Behenic Acid	0.50b		Behenic Acid	n0.00f
	Lignoceric Acid	1.41a		Lignoceric Acid	^n^0.00e
	Nervonic Acid	^n^0.00c		Nervonic Acid	0.19a
	Docosahexaenoic Acid	0.98a		Docosahexaenoic Acid	0.21f
	ΣSFA	8.47		ΣSFA	12.30
	ΣUSFA	26.92		ΣUSFA	27.55
	Total	35.40		Total	39.90

^n^ not detected, units of all compounds are percentage. Means within each column followed by the same letter are not different according to the Duncan test. Compounds in bold represent the major constituents. SFa: Saturated Fatty acids, USFa: Unsaturated Fatty Acids.

**Table 7:** Correlation coefficients among fatty acid compounds using GC on studied *Scutellaria* species.

Standards	Lauric Acid	Myristic Acid	Palmitic Acid	Oleic Acid	Linoleic Acid	Cis-11-Eicosenoic Acid	Behenic Acid	Lignoceric Acid	Nervonic Acid	Docosahexaenoic Acid
Lauric Acid	1	– 0.002^ ns^	0.622**	0.370^ ns^	0.250^ ns^	0.242^ ns^	– 0.452^ ns^	– 0.401^ ns^	– 0.321^ ns^	– 0.630**
Myristic Acid	– 0.002^ ns^	1	– 0.021^ ns^	– 0.261^ ns^	– 0.380^ ns^	0.625**	– 0.120^ ns^	0.258^ ns^	– 0.120^ ns^	0.248^ ns^
Palmitic Acid	0.622**	– 0.021^ ns^	1	0.749**	0.665**	0.263^ ns^	– 0.183^ ns^	– 0.510*	– 0.195^ ns^	– 0.370^ ns^
Oleic Acid	0.370^ ns^	– 0.261^ ns^	0.749**	1	0.982**	0.297^ ns^	0.194^ ns^	– 0.383^ ns^	– 0.431^ ns^	-0.031^ ns^
Linoleic Acid	0.250^ ns^	– 0.380^ ns^	0.665**	0.982**	1	0.201^ ns^	0.240^ ns^	– 0.320^ ns^	– 0.395^ ns^	0.014^ ns^
Cis-11-Eicosenoic Acid	0.242^ ns^	0.625**	0.263^ ns^	0.297^ ns^	0.201^ ns^	1	– 0.084^ ns^	– 0.100^ ns^	– 0.457^ ns^	0.160 ^ns^
Behenic Acid	– 0.452^ ns^	– 0.120^ ns^	– 0.183^ ns^	0.194^ ns^	0.240 ^ns^	– 0.084^ ns^	1	0.410^ ns^	– 0.527*	0.869**
Lignoceric Acid	– 0.401^ ns^	0.258^ ns^	-0.510*	– 0.383^ ns^	-0.320^ ns^	– 0.100^ ns^	0.410^ ns^	1	– 0.392^ ns^	0.660**
Nervonic Acid	– 0.321^ ns^	– 0.120^ ns^	– 0.195^ ns^	– 0.431^ ns^	-0.395^ ns^	– 0.457^ ns^	– 0.527*	– 0.392^ ns^	1	-0.494*
Docosahexaenoic Acid	– 0.630**	0.248^ ns^	– 0.370^ ns^	– 0.031^ ns^	0.014^ ns^	0.160^ ns^	0.869**	0.660**	– 0.494*	1

*Correlation is significant at the 0.05 level. **Correlation is significant at the 0.01 level. ns. Not significant.

The present study provides the first comparative characterization of crude oils obtained from the n-hexane fraction of the investigated *Scutellaria* species and demonstrates substantial interspecific variation in fatty acid composition. Unsaturated fatty acids, particularly those based on C16 and C18 chains, are widely distributed in both leaf and seed oils, whereas several rare fatty acids occur as characteristic lipid components in specific plant taxa^53^. In addition, seed oils rich in very long-chain fatty acids have attracted considerable attention due to their industrial applications^79^. These compounds may also serve as valuable chemotaxonomic markers^80^. The predominance of palmitic, oleic, and linoleic acids indicates that these species possess a lipid profile broadly consistent with other members of the Lamiaceae family^81,55^. The elevated proportions of oleic and linoleic acids in *Sc. sonowskyi* and *Sc. tomentosa* may indicate potential nutritional and pharmaceutical relevance, as these unsaturated fatty acids are associated with membrane stability, antioxidant activity, and cardiovascular benefits. In contrast, the relatively higher palmitic acid content observed in *Sc. bornmulleri* and *Sc. araxensis* suggests metabolic divergence among taxa. The strong positive correlation between oleic and linoleic acids may reflect coordinated regulation of shared biosynthetic pathways, particularly desaturation processes involved in C18 fatty acid metabolism. Conversely, the negative association between lauric acid and docosahexaenoic acid may suggest differences in carbon allocation between medium-chain and long-chain fatty acid biosynthesis. Fatty acid composition has been successfully applied as a chemotaxonomic marker in genera such as *Salvia* spp. (Lamiaceae)^57,58,16^, *Hyoscyamus* spp. (Solanaceae)^82^, *Lathyrus* spp. (Papilionoideae)^51^, and *Scutellaria galericulata* L. (Lamiaceae)^83^. Furthermore, Hamedi et al. (2017) reported 9,12,15-octadecatrienoic acid as the dominant fatty acid in the seed oil of *Scutellaria latiflora*^84^. The present findings are generally consistent with those reports while revealing clear quantitative differences among the examined *Scutellaria* species. The detection of very long-chain fatty acids such as nervonic and behenic acids is particularly noteworthy because these compounds are of increasing industrial interest and may also contribute to species discrimination within the genus. To the best of our knowledge, this study represents the first comprehensive comparative report on fatty acid composition in the investigated *Scutellaria* species. These findings expand current knowledge of lipid diversity within the genus and highlight the utility of fatty acid profiles for chemotaxonomy and future exploitation of valuable natural metabolites.

### Cluster analysis

**Fig. 5:**
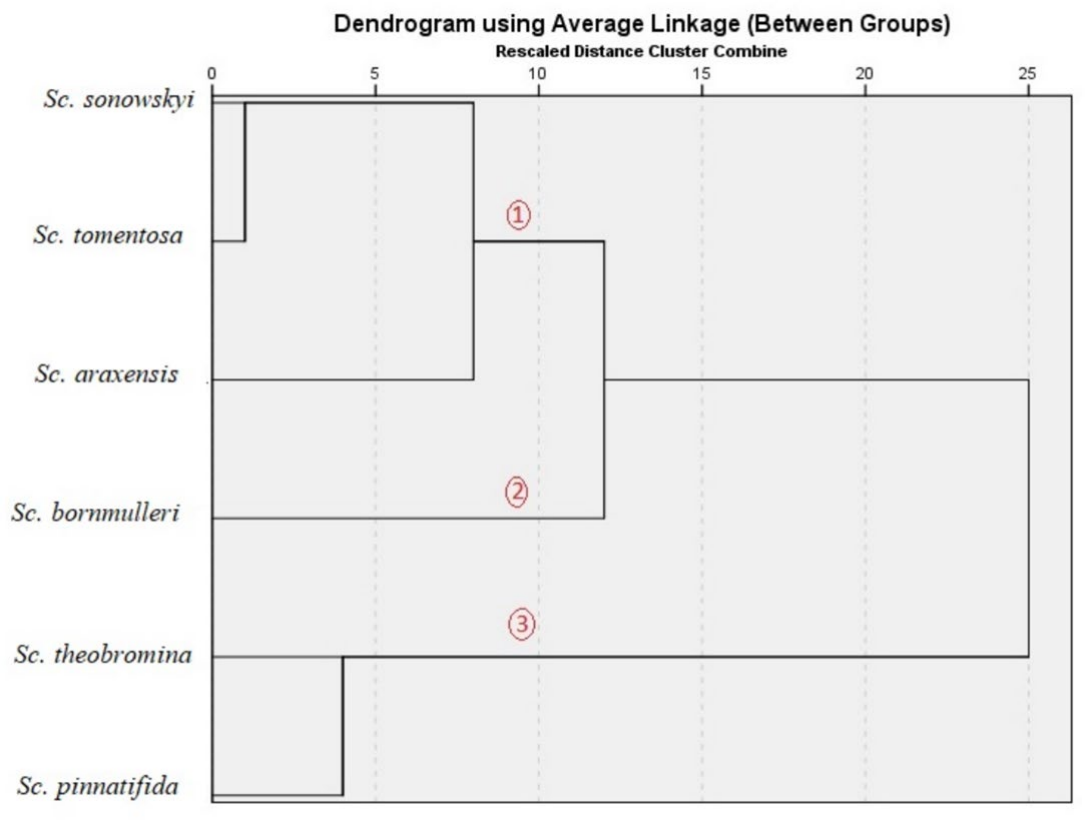
Dendrogram resulting from cluster analysis of 6 *Scutellaria* species based on 28 chemical compounds.

The hierarchical clustering analysis (HCA) generated a dendrogram with clear separation among the six investigated taxa (Fig. 5). Based on the rescaled distance coefficients, three major clusters were identified. Cluster I included *Sc. sonowskyi* and *Sc. tomentosa*, which were linked at the lowest distance level, indicating the closest affinity among all examined taxa. This subcluster was subsequently joined by *Sc. araxensis*, suggesting a relatively high similarity among these three taxa. Cluster II was represented by *Sc. bornmuelleri*, which merged with Cluster I at a higher distance coefficient. This pattern indicates that although *Sc. bornmuelleri* shares some characteristics with the taxa in Cluster I, it remains more distinct. Cluster III consisted of *Sc. theobromina* and *Sc. pinnatifida*, which formed a separate subcluster with strong internal similarity. This pair remained isolated from the remaining taxa until the final stages of clustering, demonstrating substantial divergence from Clusters I and II. Overall, the dendrogram indicates that *Sc. theobromina* and *Sc. pinnatifida* are the most distinct taxa, whereas *Sc. sonowskyi* and *Sc. tomentosa* exhibit the highest phenetic similarity. The observed clustering pattern supports clear differentiation among the studied taxa.

**Fig. 6:**
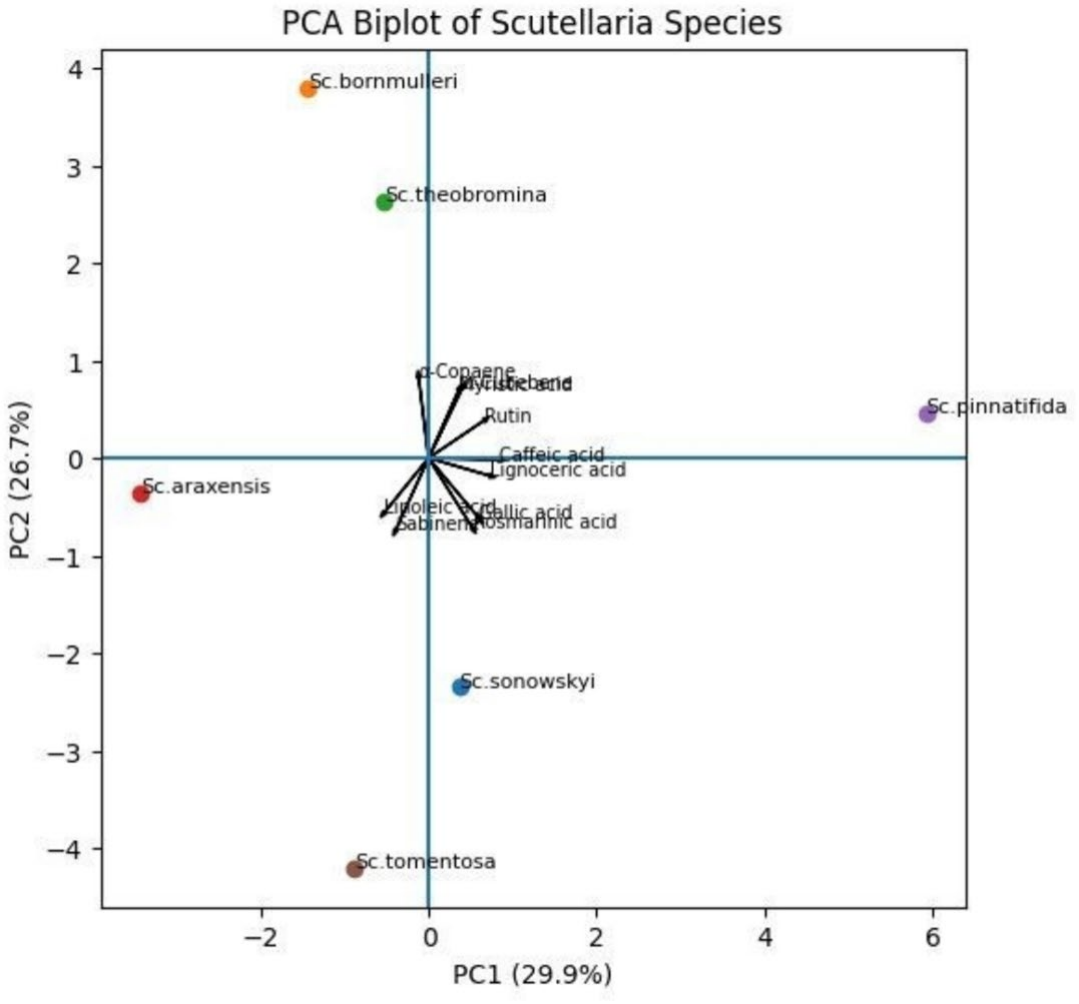
Principal component analysis (PCA) biplot illustrating the distribution of six *Scutellaria *species based on their bioactive compounds. The first two principal components explained 29.9% and 26.7% of the total variance, respectively. Arrows indicate the contribution of the most influential bioactive compounds, while points represent individual species.

Principal Component Analysis (PCA) revealed clear multivariate differentiation among the six *Scutellaria* species based on phenolic compounds, essential oil constituents, and fatty acid profiles. The first two principal components (PC1 and PC2) explained most of the total variance, indicating that these axes effectively summarized the biochemical diversity among species. PC1 was mainly associated with variation in overall metabolite abundance, particularly driven by major phenolic compounds and dominant fatty acids, whereas PC2 reflected qualitative differences in essential oil composition, separating species with distinct volatile profiles. The PCA score plot showed clear spatial segregation among species, indicating pronounced biochemical divergence (Fig. 6). This pattern, consistently supported by hierarchical clustering analysis (HCA), highlights the relevance of secondary metabolites as chemotaxonomic markers. *Sc. sonowskyi* and *Sc. tomentosa* clustered closely, suggesting strong biochemical similarity, whereas *Sc. theobromina* and *Sc. pinnatifida* were clearly separated from the remaining taxa, reflecting distinct metabolite profiles.

## Conclusion

In conclusion, the investigated *Scutellaria* species exhibited pronounced interspecific phytochemical divergence across essential oil, phenolic, and fatty acid compositions, revealing substantial metabolic heterogeneity within the genus. Essential oils were primarily characterized by sesquiterpene hydrocarbons and oxygenated terpenes, with germacrene D, trans-caryophyllene, α-pinene, and caryophyllene oxide as dominant constituents. Phenolic and fatty acid analyses further identified species-specific chemical signatures, particularly involving gallic acid, rutin, oleic acid, linoleic acid, and palmitic acid. Multivariate statistical analyses (PCA and HCA) consistently resolved the studied taxa into three major clusters, confirming the discriminatory power of phytochemical markers for taxonomic delimitation. The close clustering of *Sc. araxensis* and *Sc. tomentosa* suggests shared metabolic traits, whereas the clear separation of morphologically similar taxa such as *Sc. sosnowskyi* and *Sc. pinnatifida* highlights the superior resolution provided by chemical descriptors. Overall, the integration of comprehensive metabolite profiling with multivariate statistics offers a robust framework for chemotaxonomic classification within *Scutellaria*. Moreover, the detected chemical diversity indicates that these taxa represent valuable reservoirs of structurally diverse bioactive compounds with potential pharmaceutical, nutraceutical, and industrial applications.

## Data Availability

The datasets used and/or analyzed during the current study available from the corresponding author on reason able request.
